# Risk factors for inadequate bowel preparation before colonoscopy: a retrospective cohort study

**DOI:** 10.1186/s12876-023-02796-2

**Published:** 2023-06-13

**Authors:** Liu Shi, Foqiang Liao, Wangdi Liao, Yin Zhu, Youxiang Chen, Xu Shu

**Affiliations:** 1 Department of Gastroenterology, Digestive Disease Hospital, The First Affiliated Hospital of Nanchang University, Nanchang, 330006 Jiangxi China; 2 Department of Gastroenterology, The Affiliated Ganzhou Hospital of Nanchang University, Ganzhou, 341000 Jiangxi China

**Keywords:** Bowel preparation, Spring, Seasons, Colonoscopy, Inpatient

## Abstract

**Background:**

Colonoscopy is the standard and most effective screening tool for colonic diseases and the accuracy of colonoscopy depends on the quality of bowel preparation. The aim of this study was to analyze the risk factors for inadequate bowel preparation before colonoscopy.

**Methods:**

In this retrospective study, patients who underwent colonoscopy in 2018 and received 3 L of Polyethylene Glycol Electrolytes powder were included. They were instructed to drink 1.5 L the night before the colonoscopy and 1.5 L 4–6 h before the procedure given in doses of 250 ml every 10 min with 30 ml of simethicone given 4–6 h before the colonoscopy. Patient- and procedure-related parameters were recorded. An adequate bowel preparation was defined as all 3 segments rated 2 or 3 on the Boston Bowel Preparation scale. Risk factors for inadequate bowel preparation were identified using multivariate logistic regression analysis.

**Results:**

A total of 6720 patients were included in the present study. The mean age of these patients was 49.7 ± 13.0 years old. Inadequate bowel preparation was found in 233 (12.4%), 139 (6.4%), 131 (7%), 68 (8.6%) patients in spring, summer, autumn and winter respectively. On the multivariate analysis, male gender (OR: 1.295; 95% CI: 1.088–1.542; P = 0.005), inpatient status (OR: 1.377; 95% CI: 1.040–1.822; P = 0.025) and season (spring vs. winter, OR: 1.514; 95% CI: 1.139–2.012; P = 0.004) were the independent risk factors for inadequate bowel preparation.

**Conclusions:**

Male gender, inpatient status and spring season were the independent risk factors for inadequate bowel preparation. For patients with risk factors for inadequate bowel preparation, enhanced bowel preparation and instructions may help to optimize the quality of bowel preparation.

## Introduction

Colorectal cancer (CRC) is the third most commonly diagnosed cancer and the second leading cause of cancer death worldwide [1]. As more people have adopted western diet and lifestyles, the incidence of CRC is increasing [2]. Colonoscopy is the gold standard of CRC screening [3]. Adenoma is the precancerous lesion of CRC [4]. Colonoscopic removal of adenomatous polyps reduces mortality from colorectal cancer by up to 60%, and the risk of colorectal cancer within 10 years of colonoscopic polypectomy has been reported to be reduced to that of the general population [4, 5]. However, according a previous study, up to a fifth of lesions may be missed on colonoscopy screening [6]. The missed lesions have the potential to develop into CRC [7].

Bowel preparation plays an important role in the colonoscopy screening. Good bowel preparation can improve the quality of colonoscopy and reduces the risk of missing precancerous lesions [8, 9]. Numerous studies have identified many risk factors for inadequate bowel preparation (IBP), including diet, older age, day-prior bowel preparation, diabetes mellitus, constipation, history of abdominal operation, the use of narcotics and tricyclic antidepressants [9–12]. From 2012 to 2017, we observed that many patients underwent colonoscopy had inadequate bowel preparation quality, and the quality of bowel preparation varied in different seasons. However, there are no studies analyzing the impact of seasons on the quality of bowel preparation. To the best of our knowledge, this is the first study to analyze the season as a risk factor for inadequate bowel preparation before colonoscopy.

## Materials and methods

### Study population

This is a single-center retrospective study conducted at the Department of Gastroenterology, the First Affiliated Hospital of Nanchang University in China. Consecutive patients who received Polyethylene Glycol Electrolytes (PGE) Powder (IV) and simethicone for bowel preparation and underwent colonoscopy at the endoscopic center during 2018 were enrolled. The exclusion criteria were: (1) patients’ age under 18 years old or more than 90 years old; (2) patients who had active mental illness or were unable to give informed consent; (3) patients with colonoscopy reports without description of the quality of bowel preparation; (4) colonoscopies performed in the intensive care unit; (5) patients with incomplete demographic data. (6) pregnant or lactating females. Data collection included gender, age, chief complaint, time of colonoscopy, colonoscopy findings, season, Boston Bowel Preparation Scale (BBPS), and whether hospitalized or not. The study was approved by the Human Ethics Committee of The First Affiliated Hospital of Nanchang University. All patients provided written informed consent for colonoscopy.

### Bowel preparation

During an appointment prior to the colonoscopy, all patients would receive detailed instructions regarding dietary restrictions and corresponding preparation methods. Briefly, all patients were requested to have a low fiber diet one day prior to the colonoscopy, which included fresh peeled, pitted fruits, cooked vegetables, meat, fish, and white bread, and eating was forbidden after 6 PM the night before colonoscopy. Additionally, patients were again re-educated about the bowel preparation telephonically the day before colonoscopy [13, 14].

All patients were prescribed a split-dose preparation of 3 L PGE Powder (IV) (Beijing Staidson BioPharmaceuticals Co. Ltd., Beijing, China) plus simethicone (30mL, Zigong honghe pharmaceutical co. Ltd., Szechwan, China) given as follows: 1.5 L the night before colonoscopy, and 1.5 L given in divided doses of 250 ml every 10 min 4–6 h before the procedure with 30 ml of simethicone 4–6 h before the colonoscopy. Patients consuming other preparations were excluded from the study.

### Colonoscopy

All colonoscopies were performed by senior endoscopists with experience of more than 1000 colonoscopies. Olympus PCF-Q260AI series colonoscopies were used to perform all procedures. The procedure time was from 08:00 to 12:00 in the morning and from 14:00 to 18:00 in the afternoon. In our endoscopy center, two endoscopists are present for all colonoscopies. One endoscopist performs the colonoscopy, and the other endoscopist monitors the endoscopic images in real time and scores the quality of bowel preparation using BBPS during the procedure. The endoscopist will first learn BBPS scoring with uniform training and then the endoscopists must pass the BBPS Educational Program by obtaining a score ≥ 3 (http://www.cori.org/bbps/).

### Study endpoints

An adequate preparation was defined by all 3 BBPS segment scores ≥ 2 [15], the rating is after cleaning maneuvers are performed. The BBPS was rated from 0 (inadequate) to 3 (excellent) for each segment (left, transverse, and right) of the colon. After cleaning maneuvers are performed, the points are assigned as follows, 0: mucosa not seen due to solid stool that cannot be cleared; 1: portion of mucosa of the colon segment seen, but other areas of the colon segment not well seen due to staining, residual stool and/or opaque liquid; 2: minor amount of residual staining, but mucosa of colon segment seen well; 3: entire mucosa of colon segment seen well [16]. The right colon included the cecum and the ascending colon; the left colon consisted of the descending colon, sigmoid colon, and rectum. The transverse colon segment included the hepatic and splenic flexures. The overall score for the BBPS was the sum from all three segments, ranging from 0 (completely unprepared) to 9 (excellent).

### Statistical analysis

The variables were presented as mean ± standard deviation (SD) or proportion, as appropriate. The differences in baseline characteristics between the adequate bowel preparation and inadequate bowel preparation groups were assessed using Student’s t-test for continuous variables, and chi-square test or Fisher’s exact test for categorical variables, as appropriate. Univariate analysis was performed to assess the risk factors associated with inadequate bowel preparation, and those with a P-value of < 0.20 were incorporated into the multivariate analysis. The results were presented as odds ratios (OR) with 95% confidence intervals (95% CI). P < 0.05 was considered to be statistically significant. Statistical analyses were performed using IBM SPSS Statistics for Windows (version 23.0).

## Results

### Patient characteristics

**Fig. 1:**
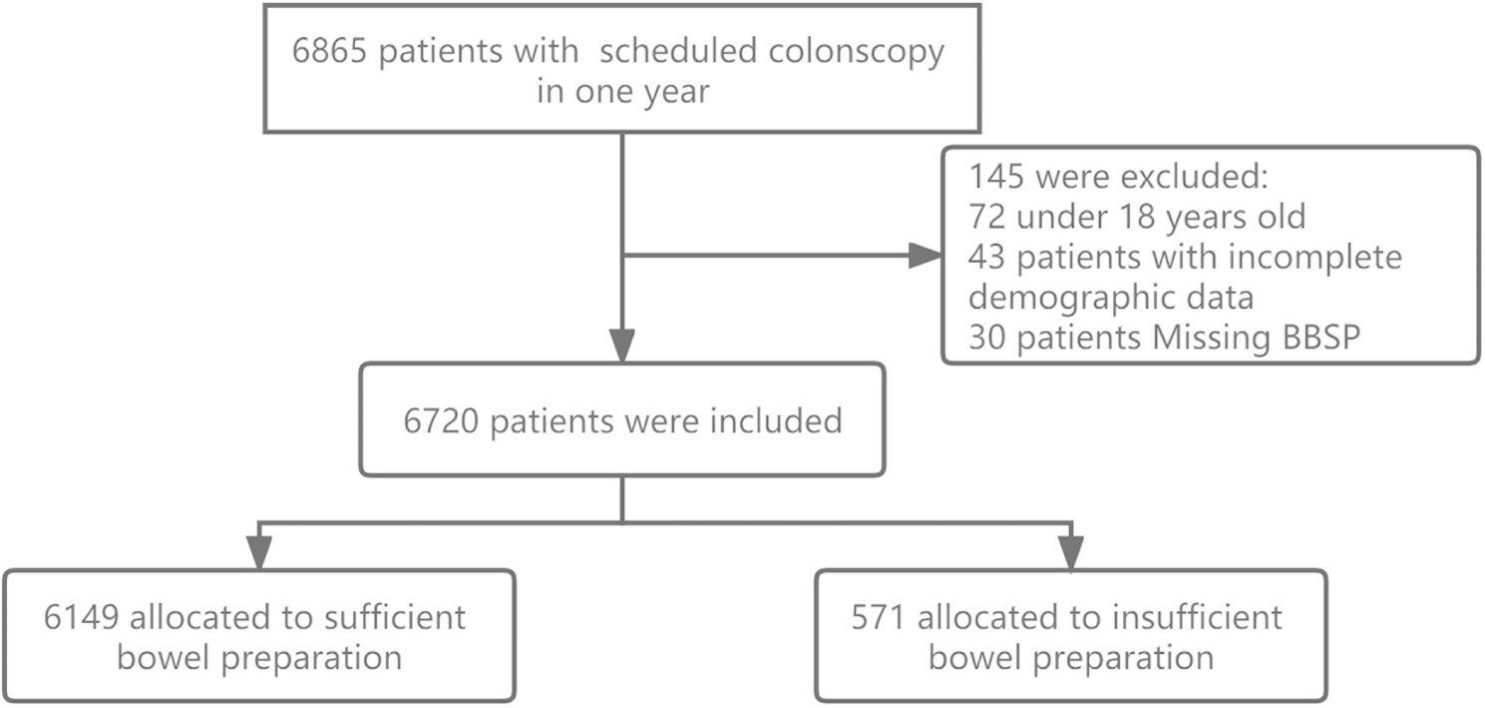
Flowchart of patients included in the present study

During the study period, a total of 6865 patients underwent colonoscopy (same patient undergoing colonoscopy more than once in the same season were only recorded once, while same patient undergoing colonoscopy in different season were recorded as different patients). 72 patients were excluded as the age were under 18 years old or more than 90 years old, 43 patients had incomplete demographic data and the quality of bowel preparation was not reported in 30 patients (Fig. 1). Finally, 6720 patients were included for analysis in this study. The mean age of the patients was 49.7 ± 13 years. 3467 (51.6%) patients were men. The highest number of colonoscopies were performed in summer (2164/6720, 32.2%), followed by spring (1883/6720, 28.0%), autumn (1882/6720, 28.0%) and winter (791/6720, 11.8%). The detailed baseline characteristics are presented in Table 1.

**Table 1:** Baseline characteristics

Patients, n		6720
Age (mean ± SD)		49.7 ± 13.0
Male		3467 (51.6%)
Chief complaint		
	Constipation	397 (5.9%)
	Abdominal pain	2207 (32.8%)
	Diarrhea	524 (7.8%)
	Health examination	3193 (47.5%)
	Others	399 (6.0%)
Time for colonoscopy		
	Morning	2885 (42.9%)
	Afternoon	3835 (57.1%)
Season		
	Spring	1883 (28.0%)
	Summer	2164 (32.2%)
	Autumn	1882 (28.0%)
	Winter	791 (11.8%)

### Outcome of colonoscopy

Inadequate bowel preparation was observed in 571(8.5%) patients. 3835 (57.1%) patients had colonoscopy in the afternoon, while 2885 (42.9%) patients underwent colonoscopy in the morning. Inadequate bowel preparation was found in 233 (12.4%), 139 (6.4%), 131 (7.0%), 68 (8.6%) patients in spring, summer, autumn and winter seasons respectively. Positive findings were detected in 3470 colonoscopies, with some patients having multiple positive findings (Table 2).

**Table 2:** Outcome of colonoscopy

Patients, n		6720
Total BBPS score		6.0 ± 0.8
Right-sided colon		2.0 ± 0.3
	BBPS = 0	35 (0.5%)
	BBPS = 1	385 (5.7%)
	BBPS = 2	6069 (90.3%)
	BBPS = 3	231 (3.5%)
Transverse colon		2.0 ± 0.3
	BBPS = 0	18 (0.3%)
	BBPS = 1	257 (3.8%)
	BBPS = 2	6140 (91.4%)
	BBPS = 3	305 (4.5%)
Left-sided colon		2.0 ± 0.4
	BBPS = 0	24 (0.4%)
	BBPS = 1	210 (3.0%)
	BBPS = 2	5929 (88.0%)
	BBPS = 3	577 (8.6%)
The quality of bowel preparation		
	Adequate*	6149 (91.5%)
	Inadequate	571 (8.5%)
Colonoscopic findings		
	Adenoma	1046 (15.6%)
	Hyperplastic polyps	1866 (27.8%)
	Enteritis	517 (8.7%)
	Carcinoma	129 (1.9%)
	No abnormalities	3252 (48.4%)
	Others	183 (2.7%)

*Adequate bowel preparations were defined as all 3 segment scores 2 or 3

### Univariate and multivariate analysis

The timing, indications for colonoscopy and colonoscopic findings were similar between the adequate bowel preparation and inadequate bowel preparation groups. The univariate analysis revealed male, age ≥ 50 years, season and inpatient status to be associated with inadequate bowel preparation (Table 3).

**Table 3:** Univariable analysis for inadequate bowel preparation as the primary outcome

		Adequate bowel preparation (n = 6149)	Inadequate bowel preparation (n = 571)	P-value	OR(95%CI)
Gender				0.002	1.280 (1.080–1.527)
	Female	3009 (48.9%)	244 (42.7%)		
	Male	3140 (51.1%)	327 (57.3%)		
Age				0.045	1.193 (1.004–1.417)
	< 50	3059 (49.7%)	259 (45.4%)		
	≥ 50	3090 (50.3%)	312 (54.6%)		
Time for colonoscopy				0.161	0.884 (0.744–1.050)
	Morning	2624 (42.7%)	261 (45.7%)		
	Afternoon	3525 (57.3%)	310 (54.3%)		
Season				<0.001	0.817 (0.747–0.893)
	Spring	1650 (26.8%)	233 (40.8%)		
	Summer	2025 (32.9%)	139 (24.3%)		
	Autumn	1751 (28.5%)	131 (22.9%)		
	Winter	723 (11.8%)	68 (12.0%)		
Chief complaint				0.459	0.970 (0.893–1.052)
	Constipation	361 (5.9%)	36 (6.3%)		
	Abdominal pain	2014 (32.8%)	193 (33.8%)		
	Diarrhea	482 (7.8%)	42 (7.4%)		
	Health examination	2941 (47.8%)	252 (44.1%)		
	Others	351 (5.7%)	48 (8.4%)		
Colonoscopic findings				0.326	1.026 (0.975–1.08)
	Adenoma	974 (15.8%)	72 (12.6%)		
	Hyperplastic polyps	1702 (27.7%)	164 (28.7%)		
	Enteritis	470 (7.6%)	47 (8.2%)		
	Carcinoma	113 (1.8%)	16 (2.8%)		
	No abnormalities	2972 (48.3%)	280 (49.0%)		
	Other	168 (2.7%)	15 (2.6%)		
Hospitalization				0.005	1.449 (1.118–1.877)
	Outpatient	5576 (90.7%)	497 (87.0%)		
	Inpatient	573 (9.3%)	74 (13.0%)		

Multivariate analysis included factors with P < 0.20 on univariate analysis. We found male subjects (OR: 1.295; 95% CI: 1.088–1.542; P = 0.005), inpatient status (OR: 1.377; 95% CI: 1.040–1.822; P = 0.025) and season were the independent risk factors for bowel preparation.

Considering winter as the reference point, patients undergoing colonoscopy in spring had worse bowel preparation (OR: 1.514; 95% CI: 1.139–2.012; P = 0.004), while patients receiving colonoscopy in summer had better bowel preparation (OR: 0.738; 95% CI: 0.546–0.948; P = 0.050) (Table 4).

**Table 4:** Multivariable analysis for inadequate bowel preparation as the primary outcome

Risk factor	Regression coefficient	Adjusted OR (95% CI)	*P*-value
Male Gender	0.259	1.295 (1.088–1.542)	0.005
Age ≥ 50	0.137	1.147 (0.963–1.366)	0.124
Inpatient status	0.320	1.377 (1.040–1.822)	0.025
Spring Season	0.451	1.514 (1.139–2.012)	0.004
Summer Season	0.303	0.738 (0.546–0.948)	0.050
Autumn Season	-0.233	0.793 (0.584–1.076)	0.136
Winter Season	…	1 (ref)	…
Afternoon colonoscopy	-0.063	0.939 (0.779–1.131)	0.506

## Discussion

Our retrospective study of 6720 subjects undergoing colonoscopy in 2018 showed that male subjects was an independent risk factor for inadequate bowel preparation (OR: 1.295; 95% CI: 1.088–1.542; P = 0.005). It may be due to the difference in the working environment and living habits of men and women, and also because male patients may be less compliant with the instructions for bowel preparation [17].

Perhaps the most important finding we identified in this study was that the season was an independent risk factor of inadequate bowel preparation. Using winter as a reference point, patients in spring had worse bowel preparation (OR: 1.514; 95% CI: 1.139–2.012; P = 0.004), while patients in summer had better colon preparation (OR: 0.738; 95% CI: 0.546–0.948; P = 0.050). The exact reason for this observation is not known. However, different seasons have different climates, and people’s activities are also different. We hypothesized that it may be people’s different activities in different season contribute to the quality of bowel preparation varied in different seasons. Summer is the hottest season. Hence, in summer, people’s activities are more frequent than other seasons. Increased activities promote intestinal peristalsis and facilitate the bowel emptying [18]. In the study area, there was also more rain in spring, and it often continued to rain. Therefore, people may have less activities in spring than in winter. In addition, fewer fresh vegetables in spring may also affect the quality of bowel preparation. However, the evidence is limited and further studies are needed to determine the reasons for inadequate bowel preparation in spring. In China, because there are many traditional festivals in winter, including New Year’s Day and Spring Festival, most people choose to get together with their relatives and friends at home, colonoscopies in winter were obviously fewer than other seasons.

Age ≥ 50 years was associated with inadequate bowel preparation on univariate analysis (OR: 1.19; 95% CI: 1.004–1.417; P = 0.045). Previous study indicated that decreased tolerance and slow gastrointestinal motility could contribute towards inadequate bowel preparation in the elderly population [18]. However, in this study, age ≥ 50 years was not an independent risk factor on multivariate analysis (P = 0.124).

In the current study, we also found inpatients had a worse colon preparation (OR: 1.377; 95% CI: 1.040–1.822; P = 0.025). Previous studies have also found that a high proportion of hospitalized patients undergoing colonoscopy had inadequate bowel preparation [19]. This may due to the proportion of inpatients with other diseases, which are risk factors for bowel preparation, was higher. Besides, hospitalized patients are less mobile compared with outpatients which may have contributed to inadequate bowel preparation. Therefore, inpatients should be provided with aggressive bowel preparation regimens and encouraged to increase their physical activity prior to colonoscopy.

There are some limitations of this study. First, the present study was a single-center retrospective study. The findings of the present study need to be validated by multicenter prospective studies. Second, multiple patient-related factors such as body mass index (BMI), patients’ education and history of colon preparation, comorbidities such as diabetes mellitus, medication history were not recorded in this study. But, whether BMI and history of colon preparation affect the bowel preparation is still not clear [9, 12, 20]. Third, since we selected polyethylene glycol electrolyte (PGE) powder (IV) and simethicone for bowel preparation, the applicability of this result to other preparations and different countries needs to be further verified. However, the number of subjects included in this study was large and no previous articles have looked at the season as a risk factor for inadequate cleansing for colonoscopy.

In conclusion, male subjects, inpatient status and spring season were the independent risk factors for inadequate bowel preparation. For patients with risk factors for inadequate bowel preparation, enhanced bowel preparation and instructions may help to optimize the quality of bowel preparation.

## Data Availability

The datasets generated and/or analysed during the current study are not publicly available due to protect the privacy of the patient, but are available from the corresponding author on reasonable request.

## List of Abbreviations

CRC: Colorectal cancer
IBP: Inadequate bowel preparation
PGE: Polyethylene Glycol Electrolytes
BBPS: Boston Bowel Preparation Scale
SD: Standard deviation
OR: Odds ratios
CI: Confidence intervals
BMI: Body mass index
